# Expression of insulin‐like growth factor‐1 receptor in circulating tumor cells of patients with breast cancer is associated with patient outcomes

**DOI:** 10.1002/1878-0261.12114

**Published:** 2017-11-16

**Authors:** Maria Spiliotaki, Dimitris Mavroudis, Maria Kokotsaki, Eleni‐Kyriaki Vetsika, Ioannis Stoupis, Alexios Matikas, Galatea Kallergi, Vassilis Georgoulias, Sofia Agelaki

**Affiliations:** ^1^ Laboratory of Translational Oncology School of Medicine University of Crete Heraklion Greece; ^2^ Department of Medical Oncology University General Hospital of Heraklion Greece

**Keywords:** breast cancer, circulating tumor cells, E‐cadherin, IGF1R

## Abstract

In patients with breast cancer, markers of aggressiveness such as dysregulation of the insulin‐like growth factor receptor (IGF1R) system and E‐cadherin loss are commonly observed. Reduced IGF1R expression is correlated with decreased E‐cadherin levels and increased cell motility. We assessed IGF1R and E‐cadherin expression in circulating tumor cells (CTCs) in patients with breast cancer. Peripheral blood mononuclear cells of early (*n* = 87)‐ and metastatic (*n* = 126)‐stage breast cancer patients (obtained prior to adjuvant and first‐line chemotherapy) were evaluated using double immunofluorescence (IF) staining for cytokeratin (CK) and IGF1R. Triple IF using CK, IGF1R, and E‐cadherin antibodies was performed in selected CTC(+) patients. IGF1R(+) CTCs were more frequently observed in early disease than in metastatic disease (86% vs 68% of CTCs, *P* = 0.04) stage, whereas IGF1R(−) CTCs were more common in metastatic than in early disease (32% vs 14% of CTCs, *P* = 0.002). 100% of CTC(+) patients with early disease, compared to 79% of those with metastatic disease, harbored IGF1R(+) CTCs (*P* = 0.007). Patients with early disease and exclusively IGF1R(+) CTCs had longer disease‐free (*P* = 0.02) and overall survival (*P* = 0.001) compared to patients with both IGF1R(+) and IGF1R(−) CTC populations. 67% of early‐stage CTC(+) patients evaluated had exclusively IGF1R(+)/E‐cadherin(+) CTCs, 33% also had IGF1R(−)/E‐cadherin(−) CTCs, and none had exclusively IGF1R(−)/E‐cadherin(−) CTCs compared to 17%, 75%, and 8% of metastatic patients, respectively (*P* = 0.027). Similarly, in paired samples of patients with early disease that progressed to metastatic disease, the proportion of IGF1R(+)/E‐cadherin(+) CTCs was reduced and IGF1R(−)/E‐cadherin(−) CTCs were increased in the metastatic stage compared to early disease stage. IGF1R(+) CTCs are commonly detected in breast cancer, and their frequency decreases in the metastatic disease stage. IGF1R(+)/E‐cadherin(+) CTCs also decrease in metastatic patients. IGF1R(+) CTCs are associated with favorable outcomes in early disease stage, suggesting that IGF1R expression is correlated with reduced metastatic potential in breast cancer.

**Abbreviations**
ARIOL system: automated image analysis system
CK: cytokeratin
CTCs: circulating tumor cells
IGF1R: insulin‐like growth factor‐1 receptor
PBMCs: peripheral blood mononuclear cells

## 1 Introduction

A significant body of evidence illustrates the important role of insulin‐like growth factor signaling system in a variety of human cancers including breast cancer (Pollak *et al*., 2004). Ligand‐induced activation of the insulin‐like growth factor‐1 receptor (IGF1R) leads to a series of phosphorylation events that activate PI3‐K and MAPK signaling pathways, resulting in cellular proliferation and inhibition of apoptosis (LeRoith and Roberts, 2003), whereas blockade of IGF1R signaling significantly inhibits cellular proliferation (Arteaga *et al*., 1989; Cullen *et al*., 1990). A number of strategies have been developed to interfere with the IGF1R pathway, some of which have entered clinical trials (Haluska *et al*., 2007; Ma *et al*., 2013; Rowinsky *et al*., 2007). Therefore, further understanding of the pattern of IGF1R expression in breast cancer and its potential impact on prognosis may be useful as these agents are being developed.

Older studies using ligand‐binding assays demonstrated lower levels of IGF1R expression in benign lesions and normal breast tissue compared with their malignant counterparts (Peyrat *et al*., 1988; Railo *et al*., 1994). In another report, IGF1R immunoreactivity was evident in the majority of breast carcinomas and was correlated with estrogen receptor (ER) expression (Happerfield *et al*., 1997). In the same study, normal mammary epithelium also demonstrated high intensity labeling, often at levels similar to carcinoma. Schnarr *et al*. (2000) also showed that insulin receptor substrate‐1 (IRS‐1) and IGF1R are expressed at high levels in control tissues and in well and moderately differentiated carcinomas, but at significantly lower levels in poorly differentiated breast cancers. Conflicting results have been also reported on the significance of IGF1R expression as a determinant of prognosis in breast cancer. Several researchers have suggested that high IGF1R levels may serve as a predictor of early disease recurrence (Peiro *et al*., 2011; Turner *et al*., 1997), whereas others have considered high IGF1R values as a marker of favorable prognosis (Aaltonen *et al*., 2014; Fu *et al*., 2011; Lee *et al*., 1998; Papa *et al*., 1993; Yerushalmi *et al*., 2012).

It is well established that the acquisition of a metastatic phenotype by breast cancer cells may be related to deactivation of adherence‐type cell junctions, structured around transmembrane cadherin proteins (Birchmeier *et al*., 1993). Cadherins are calcium‐dependent cell–cell adhesion molecules that mediate homotypic interactions among cells and modulate tissue morphogenesis (Takeichi, 1995). E‐cadherin is frequently expressed in breast epithelial cells and its presence is related to a nonmetastatic phenotype (Frixen *et al*., 1991). Moreover, loss or downregulation of E‐cadherin has been observed in several metastatic breast cancer cell lines and breast carcinomas (Sommers *et al*., 1994) and has been correlated with an epithelial‐to‐mesenchymal (EMT) phenotype (Thiery and Sleeman, 2006). In addition, clinical studies have shown that reduced or impaired E‐cadherin expression in breast cancer is associated with poor prognostic indicators such as larger tumor size, higher histological grade, development of distant metastases, ER‐negative tumors as well as decreased disease‐free and overall survival (Singhai *et al*., 2011). Several reports have shown an interaction between E‐cadherin and IGF1R at the points of cell–cell contacts which promotes cell–cell adhesion (Guvakova and Surmacz, 1997). Moreover, Pennisi *et al*. (2002) showed that reduced expression of IGF1R in MCF‐7 breast cancer cells was associated with decreased expression of E‐cadherin and increased cell motility.

Circulating tumor cells (CTCs) are purported intermediates of metastasis, through which the primary tumor is considered to ‘seed’ the metastatic site. The detection of CTCs has been established as an independent poor prognostic marker in both early and metastatic breast cancer (Cristofanilli *et al*., 2004; Rack *et al*., 2014). CTCs are a heterogeneous population of cells derived from the primary tumor and metastatic sites that could present the genetic landscape of all cancerous lesions of a given patient. In this context, evaluation of the phenotypic and molecular characteristics of CTCs could provide significant prognostic and predictive information and could guide treatment selection.

In the present study, we investigated the expression of IGF1R on CTCs of early and metastatic breast cancer patients using immunofluorescence microscopy. We also evaluated the coexpression of E‐cadherin and IGF1R in a subgroup of CTC‐positive breast cancer patients. Our results indicate that IGF1R expression in CTCs is frequently encountered in breast cancer and their incidence decreases in metastatic disease. Moreover, the incidence of IGF1R(+)/E‐cadherin(+) CTCs also decreases in metastatic patients. Importantly, we show that IGF1R expression in CTCs is associated with improved prognosis in early breast cancer.

## 2 Materials and methods

### 2.1 Patients

Women with early (*n* = 87) and metastatic (*n* = 126) breast cancer were entered in this study. Peripheral blood was collected before the initiation of adjuvant or first‐line chemotherapy from patients with early or metastatic disease, respectively. Peripheral blood was also drawn from healthy female donors (*n* = 15) who had neither known illness at the time of sampling nor any history of malignant disease to ensure the specificity of the methods used. All patients and healthy volunteers gave their informed consent to participate in the study, which has been approved by the Ethics and Scientific Committee of our institution.

### 2.2 Cell cultures

The breast cancer cell lines MCF7, MDA‐MB‐231, MDA‐MB‐453, BT474 and the nontumorigenic MCF10A human breast cells were obtained from the American Type Culture Collection (Manassas, VA, USA). Cell lysates were prepared or cells were centrifuged on cytospins according to the procedure followed for patients’ samples, to be used as controls for CK, IGF1R, and E‐cadherin staining experiments.

MCF7, MDA‐MB‐231, MDA‐MB‐453, BT474, and MCF10A cells were cultured in the appropriate medium supplemented with 10% fetal bovine serum (FBS) (GIBCO‐BRL) and 50 μg·mL^−1^ penicillin/streptomycin. Cells were maintained in a humidified atmosphere of 5% CO_2_ in air. Subcultivation was performed with 0.25% trypsin and 5 mm EDTA (GIBCO‐BRL).

Cell lysates from MDA‐MB‐231, MDA‐MB‐453, MCF7, BT474, and MCF710A cells were used for the evaluation of IGF1R and E‐cadherin expression by immunoblotting analysis. Cytocentrifuged MCF7 cells, MDA‐MB‐231 cells, and MDA‐MB‐453 cells were used for the investigation of IGF1R and E‐cadherin expression in breast cancer cell lines by double and triple immunofluorescence experiments. All experiments were performed during the logarithmic growth phase of cells.

### 2.3 Sample collection and cytospin preparation

Twenty milliliter of blood was obtained from each patient and healthy volunteers. To avoid blood contamination by epithelial cells from the skin, all blood samples were obtained after the first 5 mL of blood was discarded. Peripheral blood mononuclear cells (PBMCs) were isolated with Ficoll‐Hypaque density gradient (*d* = 1077 g·mol^−1^) centrifugation at 670 ***g*** for 30 min. PBMCs were washed three times with phosphate‐buffered saline solution (PBS) and centrifuged at 530 ***g*** for 10 min. Aliquots of 500 000 cells were centrifuged at 700 ***g*** for 2 min on glass slides. Cytospins were dried up and stored at −80 °C. Two slides from each patient were used for staining experiments, and the results were expressed as CTCs/5 × 10^5^ PBMCs.

### 2.4 Immunoblotting analysis

Cell lysates were prepared using RIPA buffer [50 mm Tris, 0.15 m NaCl, 1% Triton X‐100, 1% sodium deoxycholate, 0.1% SDS (sodium dodecyl sulfate), 1 mm EDTA (ethylenediaminetetraacetic acid), 1 mm Na orthovanadate, 1 mm PMSF (phenylmethylsulfonyl fluoride), 25 μg·mL^−1^ leupeptin, and 25 μg·mL^−1^ aprotinin]. Protein concentrations were determined using the Bradford method. Total IGF‐IR‐β and E‐cadherin were evaluated as follows: 30 μg of cell lysates was solubilized in lysis buffer and boiled for 5 min. Equal protein aliquots were subjected to SDS electrophoresis and transferred onto nitrocellulose membrane (Schleicher & Schuell Bioscience Inc., Dassel, Germany) for 60 min at 100 V. Blots were preincubated for 1 h at room temperature in TBST (Tris‐buffered saline/Tween 20) pH 7.6 containing 5% nonfat milk (blocking buffer), washed with TBST, and incubated at 4 °C overnight, in blocking buffer with a rabbit antibody against IGF‐IR‐β (Cell Signaling Technology, Inc., Danvers, MA, USA) and a mouse antibody against E‐cadherin (clone36, BD Transduction Laboratories, San Jose, CA, USA) and α‐tubulin (Sigma‐Aldrich Co. LLC, St. Louis, MO, USA). Blots were washed with TBST and incubated with horseradish peroxidase‐linked anti‐rabbit or anti‐mouse antibody in blocking buffer for 1 h at room temperature. Immunoreactivity was detected with the Western Blotting Detection Reagents (ECL, Amersham Biosciences, Piscataway, NJ, USA), and protein molecular weights were determined using a molecular weight marker (Page Ruler Prestained Protein Ladder, Fermentas International Inc., Burlington, ON, Canada).

### 2.5 Immunostaining experiments

The expression of cytokeratins (CK) and IGF1R on cytospins prepared from breast cancer cells or PBMCs was evaluated by double immunofluorescence experiments as follows. Briefly, cytospin fixation and permeabilization was performed with ice‐cold acetone/methanol 9/1 (V/V) for 20 min at room temperature (RT), followed by incubation with blocking buffer (PBS/2% FBS) for 30 min. Cytospins were washed with phosphate‐buffered saline (PBS 1x) and incubated with IGF1R rabbit antibody (Cell Signaling Technology, Inc.) diluted 1 : 50, overnight. This was followed by incubation with the secondary Alexa 555 antibody (Molecular Probes, Inc., Eugene, OR, USA). Subsequently, cells were stained with the A45‐B/B3 mouse antibody (Micromet, Munich, Germany) detecting the expression of CK8, CK18, and CK19, diluted 1 : 100, followed by incubation with the FITC antibody (Molecular Probes, Inc.). Finally, 4′,6‐diamidino‐2‐phenylindole (DAPI) antifade reagent (Invitrogen) was added to each sample for nuclear staining.

Triple immunofluorescence for CK, IGF1R, and E‐cadherin was also performed. Briefly, PBMC cytospins were fixed as described previously and stained with the IGF1R antibody diluted 1 : 50, overnight. This was followed by incubation with the secondary Alexa 633 antibody (Molecular Probes, Inc.) diluted 1/1200. Subsequently, cells were stained with the A45‐B/B3 mouse antibody diluted 1 : 100, followed by incubation with the Alexa 555 (Molecular Probes, Inc.), diluted 1 : 3000. Afterward, cells were incubated with E‐cadherin fluorescein‐conjugated monoclonal antibody (BD Transduction Laboratories) diluted 1 : 100 for 60 min. Finally, DAPI antifade reagent (Invitrogen) was added to each sample for nuclear staining.

Double staining experiments for the detection of CK and the common leukocyte antigen CD45 were performed indicatively in samples presenting high CTC numbers. Briefly, PBMC cytospins were incubated with anti‐CD45 rabbit antibody (Santa Cruz Biotechnology, Inc., Dallas, TX, USA) for 1 h along with the corresponding secondary Alexa 555 anti‐rabbit antibody (Molecular Probes, Inc.) for 45 min, followed by the A45‐B/B3 mouse antibody for 1 h along with the corresponding secondary FITC antibody (Molecular Probes, Inc.) for 45 min. DAPI antifade reagent (Invitrogen) was added to each sample for nuclear staining. In each double and triple immunofluorescent experiment using patient samples, positive controls from cytocentrifuged MCF7 cells were prepared as previously described.

In each double and triple immunofluorescent experiment using patient samples, positive controls from cytocentrifuged MCF7 cells were prepared as previously described. Negative controls, prepared by omitting the corresponding primary antibody and adding the secondary immunoglobulin G (IgG) isotype antibody, were also included in each separate experiment. The cytomorphological criteria proposed by Meng and colleagues (i.e., high nuclear‐to‐cytoplasmic ratio and cells larger than white blood cells) were used to characterize a CK‐positive cell as a CTC (Meng *et al*., 2004). All cytospins prepared from PBMCs or cell lines were evaluated using the semiautomated ARIOL microscopy system (Genetix, New Milton, UK).

In the evaluation of the samples prepared from healthy female donors, there were no CK‐positive cells identified as CTCs in any of the samples.

### 2.6 Statistical analysis

Data were analyzed using the graphpad prism software (version 6; GraphPad Software, Inc., La Jolla, CA, USA) and spss (version 20; IBM SPSS Statistics, Armonk, NY, USA). All results are expressed as median values. Chi‐square or Fisher's exact test was used to compare the frequency of CTC phenotypes among early and metastatic breast cancer patients. Mann–Whitney test was used to compare the incidence of CTCs with different phenotypes per patient between early and metastatic disease.

In patients with early breast cancer, disease‐free survival (DFS) was defined as the time from the initiation of adjuvant treatment until the day of the first evidence of disease recurrence or death from any cause and overall survival (OS) as the time from diagnosis to death from any cause. In patients with metastatic disease, progression‐free survival (PFS) was defined as the interval between the initiation of first‐line treatment until disease relapse or death from any cause. DFS, OS, and PFS curves were generated using the log‐rank (Mantel–Cox) test. Statistical significance was defined as *P* < 0.05.

## 3 Results

### 3.1 Expression of IGF1R and E‐cadherin on breast cancer cell lines by immunoblotting analysis

The expression of IGF1R, a 95‐kDa transmembrane tyrosine kinase receptor, was initially investigated in MDA‐MB‐231, MDA‐MB‐453, MCF7, and BT474 cell lines that correspond to the basal, HER2‐positive, luminal A, and luminal B breast cancer subtypes, respectively (Neve *et al*., 2006). The nontumorigenic MCF10Α human breast cells were also evaluated. The rabbit polyclonal antibody against IGF1R used in this study detects endogenous levels of the β‐subunit of the receptor and does not cross‐react with insulin receptor. It was shown that all cell lines screened, except for MDA‐MB‐453, presented IGF1R expression (Fig. S1).

Τhe expression of E‐cadherin, a 120‐kDa transmembrane glycoprotein, was also examined in the same cell lines using the 36/E‐cadherin monoclonal antibody that recognizes the E‐cadherin cytoplasmic domain. E‐cadherin was expressed in all cell lines screened except for MDA‐MB‐231 and MDA‐MB‐453 cells (Fig. S1).

### 3.2 Characterization of IGF1R and E‐cadherin expression and localization on breast cancer cell lines by immunofluorescence analysis

**Figure 1:**
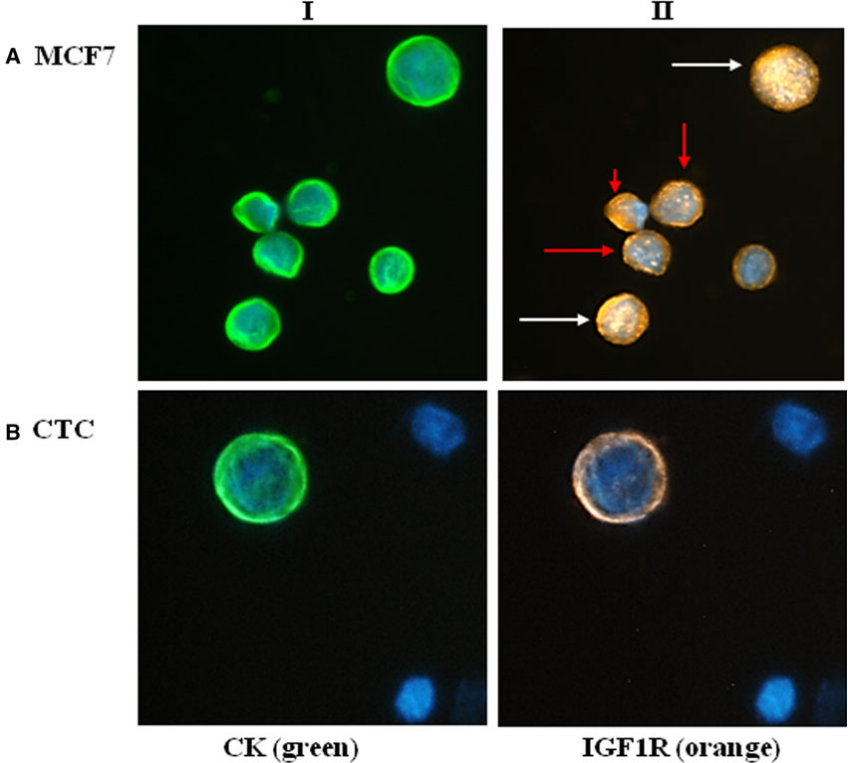
Expression of CK and IGF1R on MCF7 cells and CTCs by double immunofluorescence analysis. (AI): CK‐positive MCF7 cells. (AII): membranous (red arrows), cytoplasmic (red arrowhead), and both membranous and cytoplasmic (white arrows) staining pattern of IGF1R‐positive MCF7 cells. (BI): a CK‐positive CTC presenting membranous (BII) staining for IGF1R. Cell nuclei were stained with DAPI (blue). Images were obtained by the use of ARIOL system (×40).

**Figure 2:**
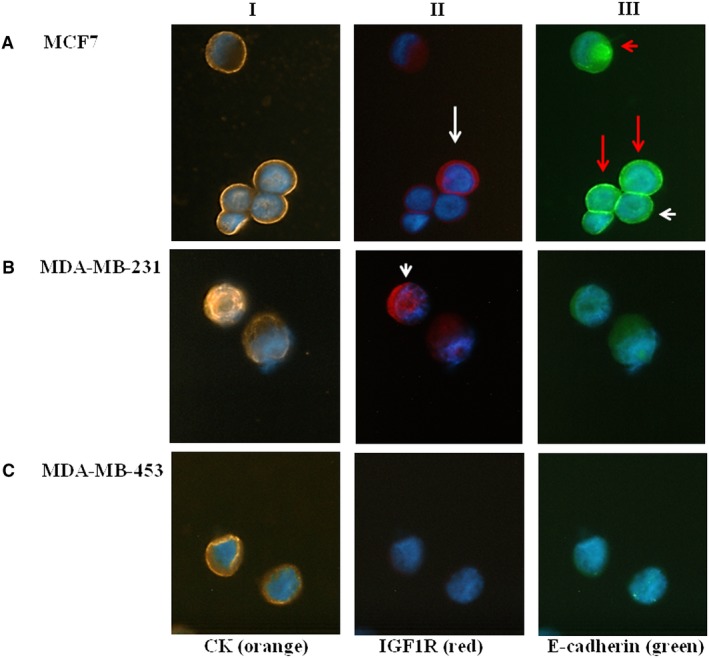
Expression of CK, IGF1R, and E‐cadherin on breast cancer cell lines by triple immunofluorescence analysis. (AI): CK‐positive MCF7 cells. (AII): membranous staining of an IGF1R‐positive MCF7 cell (white arrow). (AIII): membranous (red arrows) or cytoplasmic (red arrowhead) staining of E‐cadherin‐positive cells and loss of E‐cadherin in a MCF7 cell (white arrowhead). (BI): CK‐positive MDA‐MB‐231 cells. (BII): membranous and cytoplasmic staining of an IGF1R‐positive cell (white arrowhead) besides an IGF1R‐negative cell. (BIII): E‐cadherin‐negative cells. (CI): CK‐positive MDA‐MB‐453 cells. (CII): IGF1R‐negative (CIII) and E‐cadherin‐negative MDA‐MB‐453 cells. Cell nuclei were stained with DAPI (blue). Images were obtained by the use of ARIOL system (×40).

Insulin‐like growth factor‐1 receptor expression was evaluated in MCF7 cell cytospins using double immunofluorescence analysis. IGF1R expression was defined as positive in case of membranous, cytoplasmic, or both membranous and cytoplasmic staining pattern (Happerfield *et al*., 1997) (Fig. 1AII). The coexpression of IGF1R and E‐cadherin was evaluated in cytospins of MCF7, MDA‐MB‐231, and MDA‐MB‐453 breast cancer cells using triple immunofluorescence. In accordance with the western blot analysis, both IGF1R and E‐cadherin were expressed in MCF7 cells, and neither IGF1R nor E‐cadherin was detected in MDA‐MB‐453 cells, whereas IGF1R only was detected in MDA‐MB‐231 cells (Fig. 2). Moreover, as shown in Fig. 2AIII, heterogeneous expression and localization of E‐cadherin were observed in MCF7 cells, which displayed membranous, cytoplasmic, and/or loss of E‐cadherin staining patterns.

### 3.3 Expression of IGF1R on CTCs of early and metastatic breast cancer patients

Circulating tumor cells were detected in 28 (32%) patients with early and in 57 (45%) patients with metastatic disease; their characteristics are listed in Table 1. In early breast cancer, all CTC‐positive (+) patients harbored IGF1R(+) CTCs, seven (25%) also had IGF1R‐negative (−) CTCs, and none presented exclusively IGF1R(−) CTCs. A total of 274 CTCs were detected in patients with early disease with a median of 5 CTCs/patient (range: 1–43 CTCs); IGF1R expression was observed in 235 (86%) of these cells (Table 2). In the metastatic setting, IGF1R(+) CTCs were identified in 45 (79%) of CTC(+) patients and 12 (21%) had exclusively IGF1R(−) CTCs (*P* = 0.007, compared to patients with early disease). Of a total of 498 CTCs in metastatic patients [median of 3 CTCs/patient (range, 1–65) CTCs], 339 (68%) expressed IGF1R (Table 2) (*P*_α _= 0.04 and *P*_β _= 0.002 compared to patients with early breast cancer). There was no correlation between the detection of IGF1R(+) or IGFIR(−) CTCs and clinicopathological parameters either in early or in metastatic disease.

**Table 1:** Characteristics of CTC‐positive patients with early and metastatic breast cancer

	Early (*n* = 28)		Metastatic (*n* = 57)
Age, years median (range)	55 (36–74)	Age, years median (range)	63 (30–75)

	*N* (%)		*N* (%)
Menopausal status		Menopausal status	
Pre	10 (36)	Pre	14 (24.6)
Post	18 (64)	Post	41 (71.9)
UN	0 (0)	UN	2 (3.5)
HR status		HR status	
ER(+) and/or PR(+)	20 (71)	ER(+) and/or PR(+)	42 (73.7)
ER(−)/PR(−)	8 (29)	ER(−)/PR(−)	13 (21.1)
UN	0 (0)	UN	2 (5.2)
HER2 status		HER2 status	
(+)	1 (3.5)	(+)	11 (19.3)
(−)	26 (93)	(−)	43 (75.4)
UN	1 (3.5)	UN	3 (5.3)
Tumor size		Prior adjuvant	
T1	10 (36)	Yes	16 (28)
T2	14 (50)	No	41 (72)
T3	4 (14)	Sites of metastases	
UN	0 (0)	Nonvisceral	11 (19.2)
Grade		Visceral	6 (10.5)
I	0 (0)	Both	40 (70.2)
II	12 (42.9)		
III	9 (32.1)		
Lobular	2 (7.1)		
UN	5 (17.9)		
Number of positive nodes			
0	9 (32.1)		
1–3	10 (35.7)		
≥4	8 (28.6)		
UN	1 (3.6)		

**Table 2:** Incidence of IGF1R(+) and IGF1R(−) CTC phenotypes among CTC‐positive patients and among the total CTCs detected in early and metastatic breast cancer (double immunofluorescence analysis)

CTC phenotype (*n* %)			
CTC‐positive patients	IGF1R(+) only	IGF1R(−) only	IGF1R(+) and IGF1R(−)
Early (*n* = 28)	21 (75)	0 (0)	7 (25)
Metastatic (*n* = 57)	21 (37)	12 (21)	24 (42)

Number of CTCs detected	IGF1R(+)	IGF1R(−)
Early disease (*n* = 274)	235(86)	39 (14)
Metastatic disease (*n* = 498)	339 (68)*	139 (36)**

*P_α _= 0.04; **P_β _= 0.002 (Mann–Whitney).

To exclude the possibility of nonspecific CK staining on leukocytes, PBMC cytospins from patients with early (*n* = 10) and metastatic (*n* = 14) disease presenting high CTC numbers were evaluated for CD45 and CK staining. In the evaluation of these cytospins, no CK‐positive/CD45‐positive cells were identified.

Α representative picture of an IGF1R(+) CTC is shown in Fig. 1BI‐II.

### 3.4 Expression of IGF‐1R and E‐cadherin on CTCs in early and metastatic breast cancer

**Figure 3:**
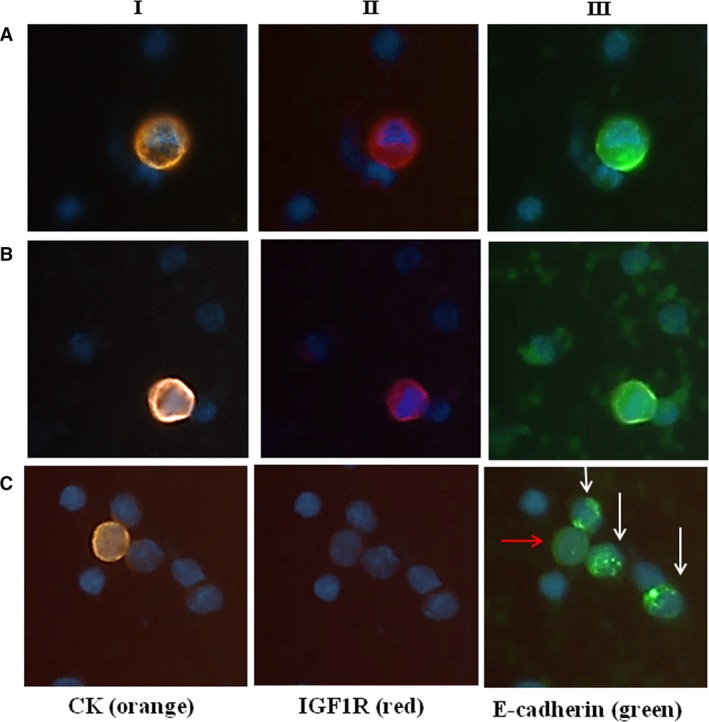
Expression of CK, IGF1R, and E‐cadherin on CTCs by triple IF analysis. (A): a CK‐positive CTC (AI) presenting cytoplasmic staining of IGF1R (AII) and cytoplasmic staining of E‐cadherin (AIII) (B): a CK‐positive CTC (BI) presenting membranous and cytoplasmic staining of IGF1R (BII) and membranous staining of E‐cadherin (BIII) (C): a CK‐positive CTC (CI), lacking IGF1R (CII) and E‐cadherin expression (CIII) (red arrow) besides E‐cadherin‐positive PBMCs (white arrows). Cell nuclei were stained with DAPI (blue). Images were obtained by the use of ARIOL system (×40).

The expression of IGF1R and E‐cadherin was indicatively evaluated in a subgroup group of early (*n* = 9) and metastatic (*n* = 12) CTC(+) patients. As shown in Fig. 3, differential staining patterns for both molecules resembling those observed in breast cancer cell lines were detected. Two different CTC phenotypes were identified according to the expression of IGF1R and E‐cadherin: IGF1R(+)/E‐cadherin(+) CTCs expressing both molecules and IGF1R(−)/E‐cadherin(−) CTCs lacking both. There were no CTCs expressing only IGF1R or E‐cadherin either in the early or in metastatic disease stage.

All patients with early disease had IGF1R(+)/E‐cadherin(+) CTCs, and in 33% of them, IGF1R(−)/E‐cadherin(−) cells were also observed (Table 3). Moreover, 67% of patients had exclusively double‐positive CTCs, whereas none had exclusively double‐negative CTCs. Of 52 CTCs detected in patients with early breast cancer, 94% were IGF1R(+)/E‐cadherin(+) (Table 3).

**Table 3:** Incidence of IGF1R(+)/E‐cadherin(+) and IGF1R(−)/E‐cadherin(−) CTC phenotypes among patients and among the total CTCs detected in early and metastatic breast cancer (triple immunofluorescence analysis)

CTC phenotype (*n* %)			
CTC‐positive patients	IGF1R(+)/E‐cadherin(+) only	IGF1R(−)/E‐cadherin(−) only	IGF1R(+)/E‐cadherin(+) and IGF1R(−)/E‐cadherin(−)
Early (*n* = 9)	6 (67)	0 (0)	3 (33)
Metastatic (*n* = 12)	2 (17)*	1 (8)*	9 (75)*

Number of CTCs	IGF1R(+)/E‐cadherin(+)	IGF1R(−)/E‐cadherin(−)
Early disease (*n* = 52)	49 (94)	3 (6)
Metastatic disease (*n* = 113)	93 (82)	20 (18)**

*P = 0.027 (Fisher's exact test) and**P = 0.014 (Mann–Whitney).

Among metastatic patients, 75% had both CTC phenotypes, 17% had exclusively IGF1R(+)/E‐cadherin(+) CTCs, and 8% had exclusively IGF1R(−)/E‐cadherin(−) CTCs (*P* = 0.027, compared to early disease, Table 3). Of a total of 113 CTCs detected in metastatic patients, 18% lacked IGF1R and E‐cadherin expression (*P* = 0.014, compared to early breast cancer, Table 3).

Four of 9 CTC(+) early breast cancer patients subsequently developed disease recurrence and they remained CTC(+) on relapse. The evaluation of these paired samples revealed that IGF1R(+)/E‐cadherin(+) CTCs were observed in all patients in both settings. However, IGF1R(−)/E‐cadherin(−) CTCs were detected in all patients on relapse compared to only 50% on initial evaluation. Among CTCs detected before the initiation of adjuvant chemotherapy, 93% were IGF1R(+)/E‐cadherin(+) compared to only 54% on relapse. Moreover, only 7% of CTCs detected in the early disease stage were IGF1R(−)/E‐cadherin(−) compared to 46% on relapse. The incidence of CTC phenotypes for each patient before the initiation of adjuvant chemotherapy and on relapse is presented in Table S1.

### 3.5 Prognostic significance of IGF1R expression in CTCs

**Figure 4:**
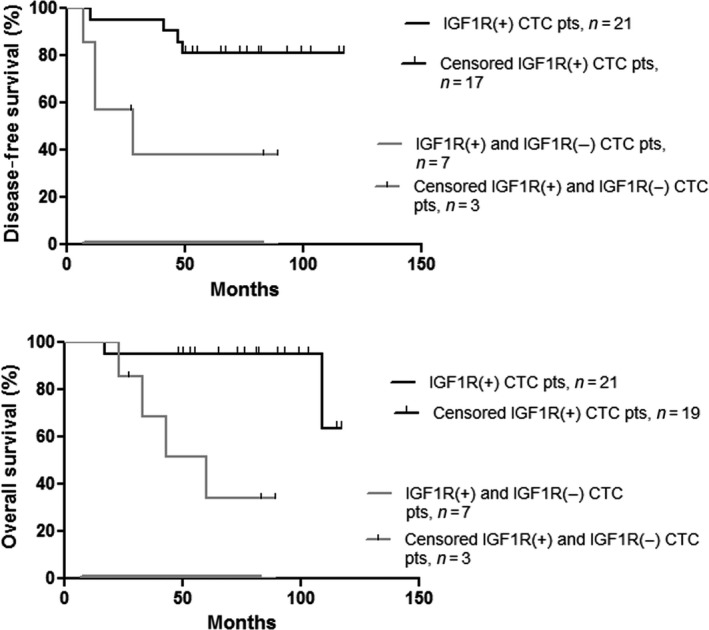
Disease‐free survival and overall survival according to IGF1R expression on CTCs in early breast cancer. Disease‐free survival and overall survival by IGF1R expression in patients (pts) with early breast cancer.

After a median follow‐up period of 74.5 months (range, 17–117), the median disease‐free survival (DFS) was 28 months (range, 7–89) for patients with early breast cancer displaying both IGF1R(+) and IGF1R(−) CTCs, while DFS was not reached for those with exclusively IGF1R(+) CTCs (log‐rank test, *P* = 0.02) (Fig. 4A). Similarly, median overall survival (OS) was 60 months (range, 23–89) for patients displaying both IGF1R(+) and IGF1R(−) CTC phenotypes, while OS was not reached for those with exclusively IGF1R(+) CTCs (log‐rank test, *P* = 0.001) (Fig. 4B). No difference in DFS or OS according to tumor size, grade, or the presence of involved lymph nodes was evident in this group of patients. Statistical analysis in metastatic patients revealed no significant differences in overall survival according to IGF1R expression (*P* = 0.57).

## 4 Discussion

The phenotypic and molecular characterization of CTCs has been suggested as a tool for the understanding of the metastatic process, the monitoring of changes in tumor biology during the course of the disease, and the refinement of patient prognosis. IGF1R has been shown to mediate cancer cell proliferation and survival, and confers resistance to cytotoxic, hormonal, and targeted therapies in breast cancer (Iams and Lovly, 2015). Recent evidence also illustrates the role of IGF1R in the maintenance of cancer stem cells, in epithelial‐to‐mesenchymal transition (EMT), and in the regulation of tumor microenvironment (Kim *et al*., 2007). In view of the development of IGF1R targeting strategies in breast cancer (Alvarez *et al*., 2010), we sought to investigate the role of IGF1R expression on CTCs of patients with early and metastatic breast cancer.

We observed that IGF1R immunoreactivity was membranous, cytoplasmic, or both membranous and cytoplasmic in control cell lines and CTCs. In accordance, Happerfield *et al*. (1997) reported similar immunohistochemical staining pattern for IGF1R in breast cancer tissues. The antibody used in our trial detects the β‐subunit of IGF1R, located on the internal side of cell membrane. As IGF1R is a transmembrane tyrosine kinase receptor, membranous localization of the staining is anticipated. Moreover, as IGF1R is translocated from the cell membrane to the cytosol, cytoplasmic IGF1R could represent a bound, internalized and thus potentially activated receptor (Fu *et al*., 2011).

Insulin‐like growth factor‐1 receptor‐expressing CTCs were detected in the majority of patients with early or metastatic breast cancer. Similarly, de Bono *et al*. (2007) also showed that IGF1R is frequently expressed in CTCs of patients with metastatic tumors, whereas Pizon *et al*. (2013) reported that 84% of patients with breast cancer had IGF1R‐expressing CTCs. Interestingly, here we report that IGF1R immunostaining is more frequently observed among CTCs in early compared to metastatic disease. Moreover, patients with early breast cancer harbored IGF1R(+) CTCs more commonly than metastatic patients, whereas none of them presented exclusively IGF1R(−) CTCs compared to 21% of metastatic patients. The reduction in the frequency of IGF1R‐expressing CTCs upon the transition from early to metastatic disease state implies a potential role for IGF1R in maintaining a less aggressive disease phenotype. In accordance with this hypothesis, *in vitro* and *in vivo* studies have shown that IGF1R expression is correlated with weak metastatic potential (Jones and Moorehead, 2008; Pennisi *et al*., 2002).

Contradictory results have been reported regarding the prognostic significance of IGF1R in breast cancer (Aaltonen *et al*., 2014; Fu *et al*., 2011; Lee *et al*., 1998; Papa *et al*., 1993; Peiro *et al*., 2011; Turner *et al*., 1997; Yerushalmi *et al*., 2012). In a large cohort of 2.871 patients with early breast cancer, IGF1R expression was correlated with good prognostic indicators and with better survival in the luminal B tumors (Yerushalmi *et al*., 2012). Similarly, IGF1R negativity was associated with worse prognosis in a cohort of mainly postmenopausal women with early disease (Aaltonen *et al*., 2014). In another series, higher IGF1R mRNA expression levels in early breast cancer were correlated with favorable clinicopathological parameters and patient prognosis (Fu *et al*., 2011). Finally, in a recent study by Engels *et al*. (2016), 66% of 2.446 postmenopausal patients with hormone receptor‐positive early breast cancer presented high IGF1R expression. In the same study, patients with high IGF1R expression treated with exemestane had significantly improved relapse‐free survival.

Importantly, in our study, patients with early disease and exclusively IGF1R(+) CTCs before starting adjuvant chemotherapy had longer DFS and OS compared to patients harboring IGF1R(−) CTCs. Our observations are in support of IGF1R expression as a favorable prognostic marker in breast cancer. They also suggest that besides the evaluation of IGF1R expression in the tumor, IGF1R expression on CTCs could provide significant prognostic information. It should be noted here that these results should be interpreted with caution in view of the exploratory nature of the analysis. Moreover, all patients included in the study had received adjuvant chemotherapy that could have influenced the prognostic information conferred by IGFIR expression on CTCs.

It is evident that a discrepancy appears to exist between data showing that IGF1R is commonly overexpressed and confers good prognosis in breast cancer and in the *in vitro* demonstration that blockade of IGF1R signaling by the use of monoclonal antibodies significantly inhibits IGF‐I‐induced proliferation of breast cancer cell lines (Arteaga *et al*., 1989; Cullen *et al*., 1990). Similar observations are derived from transgenic models, where downregulation of IGF1R results in regression of IGFIR‐induced tumors (Jones and Moorehead, 2008). In the same time, these tumors were composed primarily of cells that express epithelial markers and have weak metastatic potential, indicating that the metastatic spread probably requires additional genetic alterations. Thus, the prognostic significance of IGF1R could be similar to that of the estrogen receptor (ER) (Lee *et al*., 1998); ER mediates a mitogenic response to its ligand estradiol which can be inhibited by antiestrogens; however, its expression generally reflects well‐differentiated tumors with a favorable prognosis.

E‐cadherin plays an important role as an invasion suppressor gene/protein, as loss of its expression, abnormal function or both occur during the progression of most carcinomas and have been related to epithelial–mesenchymal transition (EMT) (Birchmeier *et al*., 1993). IGF1R colocalizes and coprecipitates with E‐cadherin in breast cancer cells (Mauro *et al*., 2001). In E‐cadherin‐positive breast cancer cells, IGF1R resulted in increased cell–cell adhesion and suppressed invasion and metastasis (Bracke *et al*., 1993; Mauro *et al*., 2001). Finally, E‐cadherin expression has been described in CTCs of lung (Hou *et al*., 2011), pancreatic (Khoja *et al*., 2013), prostate and breast cancer patients (Armstrong *et al*., 2011).

Based on the above, we selectively investigated the coexpression of IGF1R and E‐cadherin in CTCs, in a group of CTC(+) patients. We found that IGF1R and E‐cadherin were coexpressed at the single CTC level, whereas lack of IGFIR was accompanied by the absence of E‐cadherin immunostaining. Interestingly, most patients with early disease displayed exclusively IGF1R(+)/E‐cadherin(+) CTCs, whereas in the metastatic setting, most patients also harbored IGF1R(−)/E‐cadherin(−) cells besides the double‐positive population. Moreover, the analysis of paired samples obtained from patients who progressed from the adjuvant to the metastatic setting also showed that the IGF1R(+)/E‐cadherin(+) phenotype prevailed in early disease, whereas IGF1R(−)/E‐cadherin(−) CTCs were increased on relapse.

Downregulation of E‐cadherin has been associated with upregulation of mesenchymal cadherins such as N‐cadherin, EMT (Maeda *et al*., 2005) and increased invasiveness (Hazan *et al*., 1997). Moreover, in metastatic breast cancer, most CTCs display N‐cadherin expression (Armstrong *et al*., 2011). Although the small number of patients evaluated for the coexpression of IGF1R and E‐cadherin precludes firm conclusions to be drawn, the reduction in IGF1R(+)/E‐cadherin(+) CTC counts in parallel with the increase in IGF1R(−)/E‐cadherin(−) CTCs on disease progression suggests that IGF1R may preserve a less aggressive disease phenotype in breast cancer through the interaction with E‐cadherin.

In conclusion, IGF1R(+) CTCs are commonly observed in breast cancer and their incidence decreases significantly in metastatic compared to early disease. IGF1R and E‐cadherin are coexpressed at the single CTC level, and the transition from early to metastatic disease stage is associated with a reduction in the expression of both molecules. Finally, the detection of IGF1R‐expressing CTCs is associated with a favorable outcome in CTC(+) patients with early breast cancer.

The above results provide an insight into the process of metastatic dissemination in breast cancer and further underscore the significance of CTCs in the assessment of the underlying tumor biology in real time. Moreover, they support the significance of CTCs in determining patient prognosis and could be of relevance in the stratification of patients for future studies.

## Author contributions

MS participated in study design and coordination, performed the cell cultures immunofluorescence and immunoblotting experiments, analyzed the results, and drafted the manuscript. DM was involved in study design, data interpretation and participated in the preparation of the manuscript. MK participated in laboratory work and performed immunostaining experiments. EKV provided support in statistical analysis and data interpretation. IS was involved in data collection, data analysis, and interpretation. AM collected the clinicopathological data of the patients. GK participated in laboratory work. VG provided general support, was involved in data interpretation, and reviewed the manuscript. SA designed, coordinated, and supervised the study, was involved in data analysis and interpretation, and drafted the manuscript. All authors have read and approved the final manuscript.

## Supporting information


**Fig. S1.** IGF1R and E‐cadherin expression on human breast cancer cell lines by western blotting.

**Table S1.** Percentage of IGF1R(+)/E‐cadherin(+) and IGF1R(−)/E‐cadherin(−) CTC phenotypes among the total CTCs detected in early and metastatic disease stage of patients with paired samples.
