# Supplementary figures and images for: Expression of insulin‐like growth factor‐1 receptor in circulating tumor cells of patients with breast cancer is associated with patient outcomes
Source: Mol Oncol. 2017 Nov 16;12(1):21–32. doi: 10.1002/1878-0261.12114 (PMC5748482; doi:10.1002/1878-0261.12114)

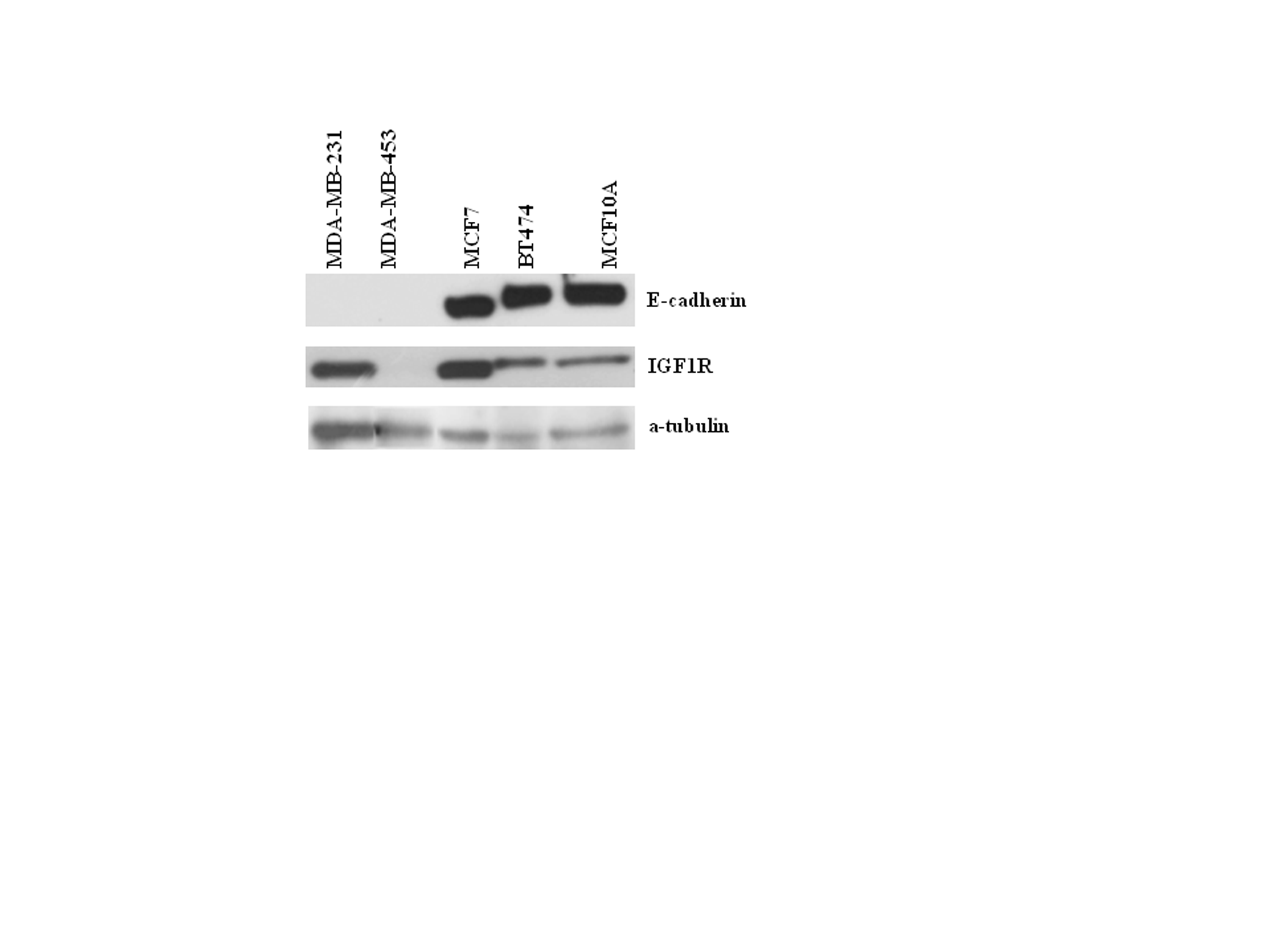

Supplement: Supplementary file 1 — Fig. S1. IGF1R and E‐cadherin expression on human breast cancer cell lines by western blotting. [file MOL2-12-21-s001.tif]
