# Supplementary material for: Expression of insulin‐like growth factor‐1 receptor in circulating tumor cells of patients with breast cancer is associated with patient outcomes
Source: Mol Oncol. 2017 Nov 16;12(1):21–32. doi: 10.1002/1878-0261.12114 (PMC5748482; doi:10.1002/1878-0261.12114)
Supplement: Supplementary file 2 [file MOL2-12-21-s002.docx]

**Supplementary Figure S1. IGF1R and E-cadherin expression on human breast cancer cell lines by western-blotting.** Total lysate (30μg) was electrophoresed by SDS-PAGE (7.5% gel) and immunoblotted for total IGF1R and E-cadherin. Western blot showing expression of IGF1R in MDA-MB-231, MCF-7, BT474 and MCF10A cell lines but not in MDA-MB-453 cells. E-cadherin was detected in MCF7, BT474 and MCF10A but not in MDA-MB-231 and MDA-MB-453 cells. A-tubulin served as a loading control.
