# Supplementary material for: Expression of insulin‐like growth factor‐1 receptor in circulating tumor cells of patients with breast cancer is associated with patient outcomes
Source: Mol Oncol. 2017 Nov 16;12(1):21–32. doi: 10.1002/1878-0261.12114 (PMC5748482; doi:10.1002/1878-0261.12114)
Supplement: Supplementary file 3 — Table S1. Percentage of IGF1R(+)/E‐cadherin(+) and IGF1R(−)/E‐cadherin(−) CTC phenotypes among the total CTCs detected in early and metastatic disease stage of patients with paired samples. [file MOL2-12-21-s003.docx]

**Supplementary Table S1.** Percentage of IGF1R(+)/E-cadherin(+) and IGF1R(-)/E-cadherin(-) CTC phenotypes among the total CTCs detected in early and metastatic disease stage of patients with paired samples

|  | **Early disease** | | | **Metastatic disease** | | |
| --- | --- | --- | --- | --- | --- | --- |
| **Pt Nu** | **Total CTCs** | **%IGF1R(+)/E-cad(+)** | **%IGF1R(-) /E-cad(-)** | **Total CTCs** | **%IGF1R(+)/E-cad(+)** | **%IGF1R(-) /E-cad(-)** |
| 1 | 9 | 89 | 11 | 10 | 50 | 50 |
| 2 | 3 | 100 | 0 | 9 | 56 | 44 |
| 3 | 9 | 89 | 11 | 3 | 67 | 33 |
| 4 | 6 | 100 | 0 | 2 | 50 | 50 |
